# Monoclonal Antibody-Based ELISA Quantification of Serum Methylglyoxal-Derived Hydroimidazolone-1

**DOI:** 10.3390/diagnostics16162593

**Published:** 2026-08-16

**Authors:** Jun Nojima, Masatsuna Tasaka, Hidetsugu Fujigaki, Sayaka Sugiura, Yasuko Yamamoto, Tetsuro Enomoto, Yushi Matuo, Kuniaki Saito

**Affiliations:** 1 Nagahama Institute for Biochemical Science, Oriental Yeast Co., Ltd., Nagahama 174-8505, Japan; nojima.jun@nisshin.com (J.N.); enomoto.tetsuro@nisshin.com (T.E.); 2 Department of Advanced Diagnostic System Development, Fujita Health University Graduate School of Medical Sciences, Toyoake 470-1192, Japan; masatsuna.tasaka@fujita-hu.ac.jp (M.T.); sayaka.sugiura@fujita-hu.ac.jp (S.S.); yama-y@fujita-hu.ac.jp (Y.Y.); matuoyushi@escaology.com (Y.M.); saitok@fujita-hu.ac.jp (K.S.)

**Keywords:** methylglyoxal-derived hydroimidazolone-1 (MG-H1), advanced glycation end products (AGEs), enzyme-linked immunosorbent assay (ELISA), diabetic kidney disease, MG-H1 specific monoclonal antibody

## Abstract

**Background/Objectives:** Methylglyoxal-derived hydroimidazolone-1 (MG-H1), an advanced glycation end product, has implications in the pathogenesis of diabetic kidney disease (DKD). Although liquid chromatography–mass spectrometry is the current gold standard for quantifying MG-H1, its overall complexity limits its utility. We developed an ELISA to measure MG-H1 using a specific monoclonal antibody. **Methods:** Competitive ELISA was used to quantify total, high-molecular-weight (HMW), and low-molecular-weight (LMW) MG-H1 in serum. The assay’s specificity was validated against structurally related compounds. Spike-and-recovery experiments were conducted to assess accuracy and precision. Serum samples from healthy controls, diabetic patients without kidney disease, and patients with DKD were analyzed (*n* = 10, 23, and 19, respectively). MG-H1’s correlation with renal biomarkers and diagnostic performance was assessed using receiver operating characteristic analyses. **Results:** The ELISA exhibited preferential reactivity toward MG-H1 compared with structurally related compounds. Spike-and-recovery experiments resulted in recovery rates ranging 108–119%. MG-H1 levels were increased in patients with DKD, although the magnitude of the changes varied among the MG-H1 forms. All MG-H1 forms correlated positively with serum creatinine and blood urea nitrogen, and negatively with estimated glomerular filtration rate. No significant correlations were observed with glycoalbumin, and only a modest association was observed between LMW MG-H1 and HbA1c. Exploratory ROC analyses suggested that all MG-H1 forms could discriminate DKD from DM, with total and HMW MG-H1 showing performance comparable to that of conventional renal function markers. **Conclusions:** This competitive ELISA enables high-throughput quantification of MG-H1 in serum and demonstrates analytical feasibility; further multicenter validation is required before clinical implementation.

## 1. Introduction

Advanced glycation end products (AGEs) represent a diverse group of compounds formed through non-enzymatic glycation reactions between reducing carbohydrates and proteins, lipids, or nucleic acids [1]. The reaction involves the formation of a Schiff base, which rearranges into a more stable Amadori product. Subsequently, these early glycation products undergo further complex chemical transformations, resulting in the formation of late-stage glycation products. AGEs accumulate naturally during aging; however, they significantly accelerate under hyperglycemic conditions, such as in diabetes mellitus [1]. Their formation is also induced by oxidative stress and inflammation. AGEs alter the structure and function of various molecules, disrupt cellular processes, and contribute to the pathogenesis of many chronic diseases, including cardiovascular disease, neurodegenerative disorders, and diabetes [2,3,4,5,6,7].

Methylglyoxal-derived hydroimidazolone-1 (MG-H1 or N(delta)-(5-hydro-5-methyl-4-imidazolone-2-yl)-ornithine) is formed through the non-enzymatic reaction of methylglyoxal (MGO) with arginine residues on proteins [8,9]. MG-H1 is formed from MG, a reactive dicarbonyl compound that is a byproduct of glycolysis [10]. MGO is known for its high reactivity and ability to modify arginine residues in proteins, resulting in structural and functional alterations [11,12]. MG-H1 is not only present in tissues under hyperglycemic conditions but also exerts potent pro-inflammatory effects, which are mediated through the receptor for advanced glycation end products (RAGE) [9,13]. MG-H1 activates inflammatory pathways through RAGE and is involved in diabetic kidney disease (DKD) as well as other syndromes. Using LC–MS/MS analysis, several studies have found that increased levels of MG-H1 in the blood of patients with DKD reflect increased glycation and renal dysfunction [14,15,16].

DKD is a microvascular complication of diabetes and the primary cause of end-stage renal disease (ESRD) [17]. Despite advances in glycemic control, a substantial residual risk of DKD progression exists. Early identification of DKD is clinically critical because renal impairment is often irreversible once advanced stages are reached. Current markers, such as serum creatinine (Cre) and estimated glomerular filtration rate (eGFR), typically reflect kidney damage only after substantial loss of renal function, limiting opportunities for timely intervention. Therefore, there is a strong need for biomarkers that enable screening of patients with diabetes for early DKD and differentiation of patients with DKD from those with diabetes without kidney disease to guide preventive strategies and optimize patient management.

Among the molecular mechanisms underlying DKD, AGE accumulation promotes renal injury through oxidative stress, inflammation, and fibrosis [1,18]. Consistently low serum MG-H1 levels are associated with a significantly reduced risk of DKD development and progression [19]. These studies indicate that circulating MG-H1 levels are associated with DKD and renal dysfunction. However, the potential utility of MG-H1 for early detection of DKD or prediction of disease progression remains to be established. Although LC–MS/MS-based measurements have provided important insights into the clinical relevance of MG-H1, this technique is not feasible for routine use in clinical settings. Therefore, a simple, high-throughput assay is needed to quantify MG-H1.

Previous studies used commercially available ELISA kits to measure MG-H1 in various pathological conditions [20,21]. However, these assays provide limited information on cross-reactivity and generally only measure protein-bound MG-H1, which limits their clinical utility. In human serum, MG-H1 predominantly exists in a protein-bound form (high-molecular-weight, HMW MG-H1), accompanied by trace amounts of low-molecular-weight (LMW) MG-H1 in various configurations [11]. The HMW fraction reflects long-term glycation of structural and circulating proteins, whereas the LMW fraction is released during proteolytic degradation of glycated proteins and is primarily excreted via the kidneys. Measurement of these MG-H1 forms has been suggested to be clinically relevant for assessing renal function and evaluating the risks of cardiovascular and neurodegenerative diseases [14]. Therefore, fraction-resolved quantification of MG-H1 (total, HMW, and LMW) might provide more comprehensive insights into metabolic stress and disease progression than conventional single-measurement approaches.

To overcome the limitations of previous immunoassays, such as cross-reactivity, an inability to selectively measure MG-H1 forms, and the complexity of LC–MS/MS, we developed a monoclonal antibody-based ELISA with high specificity for MG-H1 capable of quantifying total, HMW, and LMW fractions in serum. In this study, we selected a monoclonal antibody with high specificity for MG-H1 and low cross-reactivity to structurally related compounds, including MG-H2, MG-H3, and carboxyethyl arginine (CEA). Using this antibody, we developed and validated a competitive ELISA system for clinical application. In addition, total, HMW, and LMW MG-H1 were measured separately using this ELISA system. This assay was applied to serum samples from patients with DKD and healthy controls to evaluate its diagnostic performance and clinical relevance.

## 2. Materials and Methods

### 2.1. Materials

MG-H1, MG-H2, MG-H3, and CEA were purchased from Iris Biotech GmbH (Marktredwitz, Germany). MGO was obtained from Sigma-Aldrich (St. Louis, MO, USA). 3,3′,5,5′-Tetramethylbenzidine (TMB) was obtained from Thermo Fisher Scientific (Waltham, MA, USA). Arginine monohydrochloride, sulfuric acid, and other reagents were purchased from Nacalai Tesque, Inc. (Kyoto, Japan).

### 2.2. Ethical Approval and Sample Source

The study was approved by the Ethics Committee for Clinical Research in the Center for Research Promotion and Support at Fujita Health University (authorization numbers HM24-242 and HM21-566). It was conducted in accordance with the Declaration of Helsinki. Residual serum samples from patients with type 2 diabetes without kidney disease (DM group) or patients with DKD (DKD group) were procured from the Fujita Health University Support Office for Bioresource Research. They were collected during routine clinical examinations at Fujita Health University Hospital. Serum samples from participants without diabetes or kidney disease (control group) were obtained from the Resource Center for Health Science (RECHS) [22], which is a biobank that collects biological samples from healthy individuals who are recruited during their routine annual health checkups at participating hospitals. All samples were anonymized before analysis. Written informed consent was obtained from all participants at the time of sample collection. The serum samples were aliquoted immediately after collection and stored at −80 °C in an ultra-low-temperature freezer (MDF-DU500ZHS1, PHC Corporation, Tokyo, Japan) until analysis, with no more than one freeze–thaw cycle prior to measurement. The storage temperature was continuously monitored to ensure sample integrity.

### 2.3. Participant Classification and Clinical Data

The participants were classified into three groups: healthy controls (Ctrl), patients with type 2 diabetes without kidney disease (DM), and patients with DKD, which included those diagnosed with chronic kidney disease (CKD). Diagnostic information, which included the presence or absence of diabetes and kidney disease, was procured from the biobank records of Fujita Health University Hospital based on clinical documentation and laboratory data. The DM and DKD groups were classified based on these biobank records and were not independently adjudicated by the investigators in this study. No independent reference standard was established, and group assignment was based on the clinical diagnoses documented in the biobank records. Because anonymized residual serum samples obtained through a biobank were used, detailed clinical information on CKD stage, albuminuria status, diabetes duration, and formal exclusion of non-diabetic kidney disease was not available for all participants. Other clinical parameters, such as age, sex, Cre, blood urea nitrogen (BUN), eGFR, HbA1c, and glycoalbumin (GA), were obtained. The demographic and clinical characteristics of the study participants, including age, sex distribution, glycemic parameters, and renal function markers, together with statistical comparisons among groups, are summarized in Table 1.

### 2.4. Preparation of an Anti-MG-H1 Monoclonal Antibody and the Evaluation of Its Cross-Reactivity

Of the five monoclonal antibodies described in our previous study [23], the clone exhibiting the highest specificity for MG-H1 was identified and used for ELISA. MG-H1 exists as multiple isoforms, including MG-H2 and MG-H3 [24], and undergoes interconversion with CEA [5]. To ensure analytical specificity, the monoclonal antibodies were selected to avoid cross-reactivity with these structurally related compounds. To examine the cross-reactivity of the anti-MG-H1 antibody, structurally related compounds, including other AGEs (MG-H2, MG-H3, and CEA) and MG-H1 precursors (MGO and arginine), were evaluated as antigens. Each analyte was prepared at concentrations of 4 µM, 10 µM, and 25 µM in a dilution buffer.

### 2.5. MG-H1 Measurement by ELISA

Serum MG-H1 levels were measured using an in-house competitive ELISA method established in our laboratory (MG-H1 Competitive ELISA kit, Oriental yeast Co., Ltd., Tokyo, Japan). This assay separately quantified LMW MG-H1, HMW MG-H1, and total MG-H1. To prepare LMW and HMW MG-H1s, serum samples were passed through a 10 kDa molecular weight cut-off filter (Millipore, Burlington, MA, USA) as previously described [14]. The filtrate (lower fraction) containing low-molecular-weight compounds was used to measure LMW MG-H1, whereas the retentate (upper fraction) containing high-molecular-weight compounds was used to measure HMW MG-H1. Untreated serum samples were used for total MG-H1 without further processing. The monoclonal antibody recognizes MG-H1 in protein- and peptide-bound forms, as confirmed in our previous study [23], and reacts with authentic free MG-H1. In this study, MG-H1 species smaller than 10 kDa were categorized as LMW MG-H1, including free MG-H1 and MG-H1 bound to small peptides.

ELISA was carried out as previously described [23] with several modifications to enhance performance. To improve antibody orientation and binding efficiency, the anti-MG-H1 monoclonal antibody was biotinylated and immobilized on streptavidin-coated plates [25]. Further optimization of assay conditions was done, including incubation time. As a result, enhanced performance in the competitive reaction curve was attained with high sensitivity and minimal cross-reactivity.

The anti-MG-H1 antibody was mixed with streptavidin in a plate for 30 min. Following incubation at room temperature, the wells were washed with PBS containing 0.05% Tween 20 (PBS-T, washing buffer) three times. Next, a competitive assay was conducted by adding horseradish peroxidase-labeled MG-H1 and MG-H1 to serum samples, which were diluted 2- or 4-fold in PBS-T. After incubation at room temperature for 1 h, the wells were washed with PBS-T five times, followed by color development with substrate solution (TMB). The reaction was terminated by adding 1.0 M sulfuric acid, and the absorbance was measured at 450 nm using a microplate reader (SpectraMax Mini, Molecular Devices, San Jose, CA, USA). The concentrations of MG-H1 in serum samples were determined based on standard curves established using authentic MG-H1 as standards. The serum samples were diluted 2- or 4-fold in PBS-T based on preliminary optimization to maintain MG-H1 concentrations within the linear range of the standard curve and reduce matrix interference. Calibration was performed using authentic MG-H1 standards (Iris Biotech GmbH, Marktredwitz, Germany) to construct competitive binding curves. The HRP-labeled MG-H1 tracer was generated using the method described by Yamaguchi et al. [23], which involved covalent conjugation of MG-H1 to horseradish peroxidase.

### 2.6. Validation of ELISA

To determine the accuracy and precision of ELISA for quantifying MG-H1, spike-and-recovery experiments were conducted using human serum samples. Fixed concentrations of authentic MG-H1 (2.5, 5, and 10 μmol/L) were added to serum, and each spiked sample was analyzed in triplicate. The recovery rates were calculated by comparing the measured concentrations to the expected concentrations. Assay precision was evaluated by calculating the coefficient of variation for each concentration. According to the FDA Bioanalytical Method Validation Guidance, assay performance is generally considered acceptable when accuracy is within ±15% of the nominal concentration and precision has a coefficient of variation (CV) ≤ 15%.

Intra-assay reproducibility was assessed by measuring two serum samples with different MG-H1 concentrations (mean values: 4.45 μmol/L and 7.87 μmol/L) ten times in a single run and calculating the CV%. The limit of detection (LOD) and limit of quantification (LOQ) were determined according to IUPAC recommendations. The LOD was calculated by dividing 3.29 times the SD of the blank by the slope of the calibration curve, whereas the LOQ was calculated by dividing ten times the SD by the slope. To assess interference, serum samples were spiked with free bilirubin (0, 0.32, 0.64, 1.29, and 2.58 mg/dL), bilirubin C (0, 0.31, 0.63, 1.25, and 2.50), hemoglobin (0, 8.28, 16.56, 33.13, and 66.25), and chyle (0, 22.19, 44.38, 88.75, and 177.50 formazin turbidity units) using Interference Check A Plus (Sysmex Corporation, Kobe, Japan). OD_450_ values were measured, and the data are presented as mean ± SD of six replicate samples.

### 2.7. Statistical Analysis

Statistical analysis was performed using GraphPad Prism version 8.4.3 for Windows (GraphPad Software, San Diego, CA, USA). Data are expressed as the mean ± SD or as median and interquartile range. For the demographic and clinical variables summarized in Table 1, differences in continuous variables among groups were assessed using Welch’s ANOVA with the Brown–Forsythe correction for unequal variances and sample sizes. Pairwise group comparisons were conducted using the Games–Howell method with Holm adjustment for multiple testing. Sex distributions were assessed using the chi-square test, with pairwise group comparisons conducted using Fisher’s exact test with Holm correction. MG-H1 levels among groups were compared using the Kruskal–Wallis test followed by Dunn’s multiple-comparison test with Bonferroni correction. Correlations between MG-H1 levels and clinical parameters were determined using Spearman’s rank correlation coefficients. Receiver operating characteristic (ROC) curve analysis was conducted to evaluate the diagnostic performance of each MG-H1 form for identifying patients with DKD. Areas under the curve (AUCs) are reported with 95% confidence intervals (CIs) calculated using the Wilson/Brown method. *p* < 0.05 was considered statistically significant. Correlated ROC curves were compared pairwise using the DeLong test, implemented in R software (version 4.6.1; R Foundation for Statistical Computing, Vienna, Austria) with the pROC package. A two-sided *p* value < 0.05 was considered statistically significant.

## 3. Results

### 3.1. Baseline Characteristics of the Study Participants

The demographic and clinical characteristics of the study participants are presented in Table 1. Significant group differences were identified for age, sex distribution, HbA1c, glucose, Cre, BUN, and eGFR. Relative to the Ctrl and DM groups, the DKD group had significantly impaired renal function, with higher serum Cre and BUN levels and lower eGFR values. In addition, HbA1c and glucose levels were significantly higher in both diabetic groups than in the Ctrl group.

### 3.2. Specificity of Monoclonal Anti-MG-H1 Antibody and Validation of ELISA

**Figure 1:**
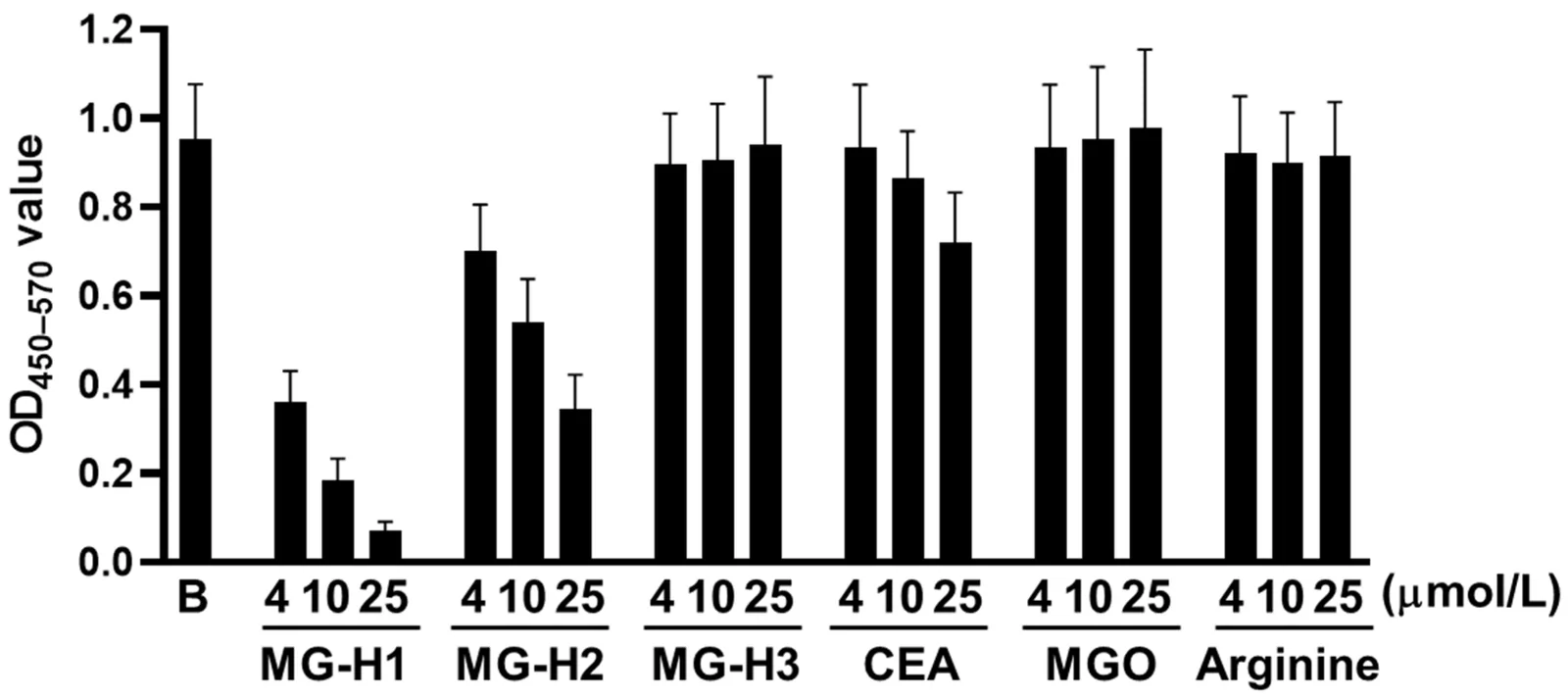
Specificity of the anti-MG-H1 antibody. OD_450–570_ values were determined for MG-H1 and structurally related compounds, including MG-H2, MG-H3, carboxyethyl arginine (CEA), methylglyoxal (MGO), and arginine. The values are shown as the mean ± standard deviation (SD) (*n* = 3). B indicates blank (no antigen).

To determine the specificity of the monoclonal anti-MG-H1 antibody, ELISA was performed using MG-H1 and structurally related compounds as antigens, including other AGEs (MG-H2, MG-H3, CEA) and MG-H1 precursors (MGO and arginine). The antibody exhibited strong reactivity toward MG-H1, with a dose-dependent decrease in OD_450_ values (Figure 1). In contrast, incubation with MG-H3, CEA, MGO, and arginine did not result in a notable reduction in OD_450_ values, indicating that the antibody has a high specificity for MG-H1. Although a decrease in OD_450_ value was also observed with MG-H2, the response was less pronounced than that with MG-H1. Because MG-H2 is not present in human serum [5,24,26], the observed cross-reactivity is not likely to be biologically relevant and may be considered negligible in the context of human applications. These results qualitatively indicate preferential recognition of MG-H1 over structurally related compounds under the tested experimental conditions. A residual response to MG-H2 was detected; however, because MG-H2 is not present in human serum, this cross-reactivity is unlikely to affect the assay’s clinical applicability.

To determine the accuracy of ELISA, spike-and-recovery experiments were conducted by adding known concentrations of authentic MG-H1 to human serum samples (Table 2). Recovery ranged from 108% to 119%, demonstrating a modest positive bias. Although recovery at the highest spike level exceeded the predefined acceptance criterion, the deviation was modest and observed only at a single concentration level. This positive bias might be attributable to matrix effects, and further investigation is warranted to clarify the underlying cause and its potential impact on absolute quantification.

Intra-assay precision was used to evaluate the reproducibility of ELISA. Two serum samples with different MG-H1 concentrations were each measured ten times within a single run. The mean MG-H1 concentrations were 4.45 μmol/L and 7.87 μmol/L in the low- and high-concentration samples, respectively. The CV was 10.40% and 14.79% for the low- and high-concentration samples, respectively. Intra-assay precision met the standard bioanalytical validation criteria, whereas recovery experiments showed a modest positive bias, particularly at the highest spike concentration.

The MG-H1 calibration curve generated using ELISA is shown in Supplementary Figure S1. Across the concentration range of 0.26 to 25.0 μmol/L, the coefficient of determination (R^2^) was 0.99, indicating reliable MG-H1 quantification within this range using the developed assay. The LOD was calculated by dividing 3.29 times the SD of the blank by the slope of the calibration curve. The mean blank absorbance was 1.51, with a standard deviation of 0.05, and the calibration curve slope was 0.50. Based on these values, the LOD for MG-H1 was 0.30 μmol/L, whereas the LOQ, defined as ten times the standard deviation of the blank divided by the slope, was 0.91 μmol/L.

Interference was evaluated by spiking serum samples with potential endogenous substances at clinically relevant concentrations. Although there were minor fluctuations in MG-H1 measurements, no significant interference was detected. Specifically, free bilirubin at concentrations up to 2.58 mg/dL, conjugated bilirubin up to 2.50 mg/dL, hemoglobin up to 66.25 mg/dL, and chyle up to 177.5 FTU did not substantially affect MG-H1 quantification (Supplementary Figure S2).

To evaluate the performance of the 10 kDa ultrafiltration procedure, we assessed the mass balance between total MG-H1 and the sum of HMW and LMW MG-H1. Mean recovery (mean ± SD) was 101.1% ± 19.3%. Despite variability among samples, the average recovery was close to 100%, suggesting that fractionation did not result in substantial systematic loss of MG-H1. Overall, the validation results indicate that although reproducibility may require minor improvement, ELISA provides acceptable analytical performance and is generally suitable for measuring MG-H1 in clinical serum samples.

### 3.3. Serum Levels of Total, LMW, and HMW MG-H1 in the Control, DM, and DKD Groups

**Figure 2:**
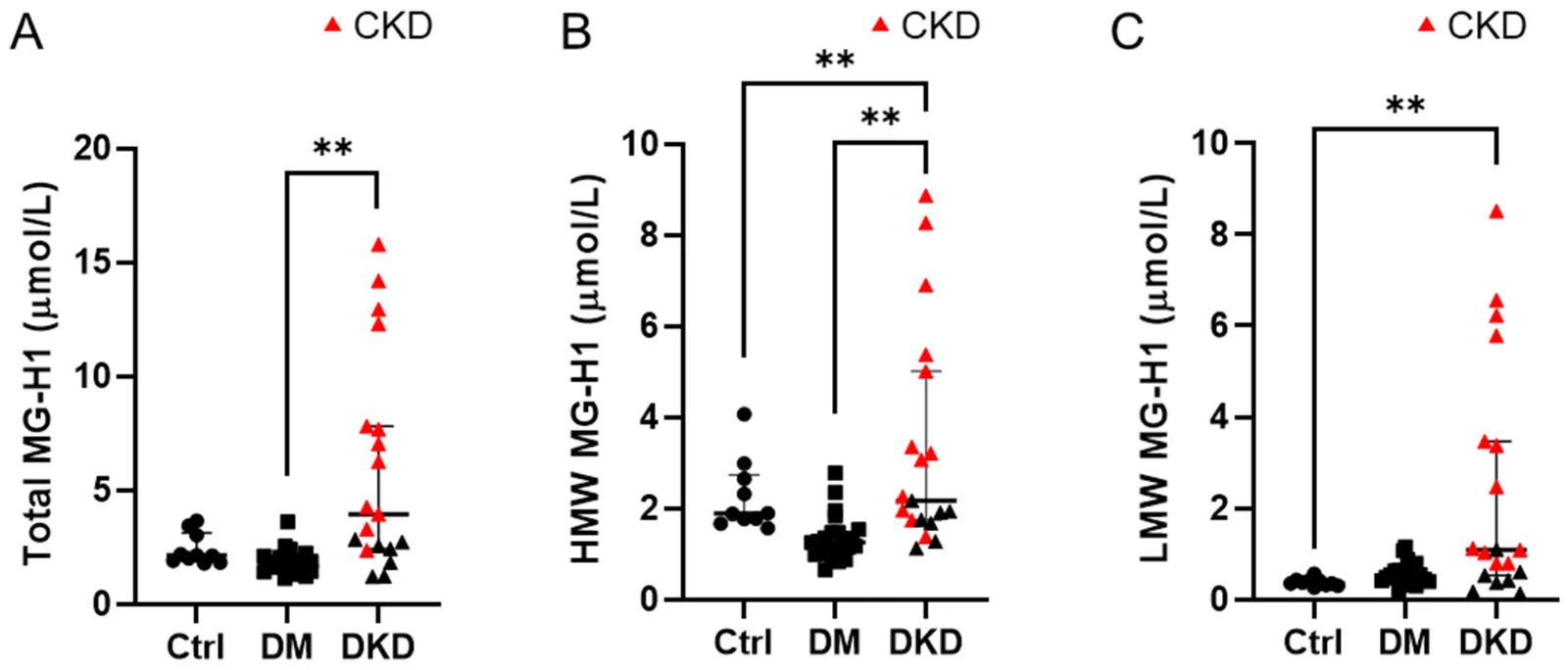
Individual levels of total (**A**), high-molecular-weight (HMW, (**B**)), and low-molecular-weight (LMW, (**C**)) MG-H1 in the control (Ctrl), diabetes without kidney disease (DM), and diabetic kidney disease (DKD) groups. Data are presented as the median and interquartile range. Red dots indicate patients with chronic kidney disease (CKD), a subset of the DKD group (*n* = 12). In this study, patients with CKD were analyzed as part of the DKD group. Statistical analysis was performed using the Kruskal–Wallis test followed by Dunn’s test with Bonferroni’s correction for pairwise comparisons. ** *p* < 0.01.

Figure 2 shows the individual measurements of total (A), HMW (B), and LMW MG-H1 (C) in the Ctrl, DM, and DKD groups. Total MG-H1 levels were significantly higher in the DKD group than in the DM group. The HMW MG-H1 levels were significantly higher in the DKD group than in both the Ctrl and DM groups. Similarly, LMW MG-H1 levels were significantly increased in the DKD group compared with the Ctrl group. Among all groups, the DKD group consistently exhibited the highest levels of total, HMW, and LMW MG-H1. Furthermore, patients with CKD tended to show elevated levels across all MG-H1 forms, further supporting the association between MG-H1 accumulation and renal dysfunction.

### 3.4. Correlation Between Three Types of MG-H1 Levels and Renal Function Markers

**Figure 3:**
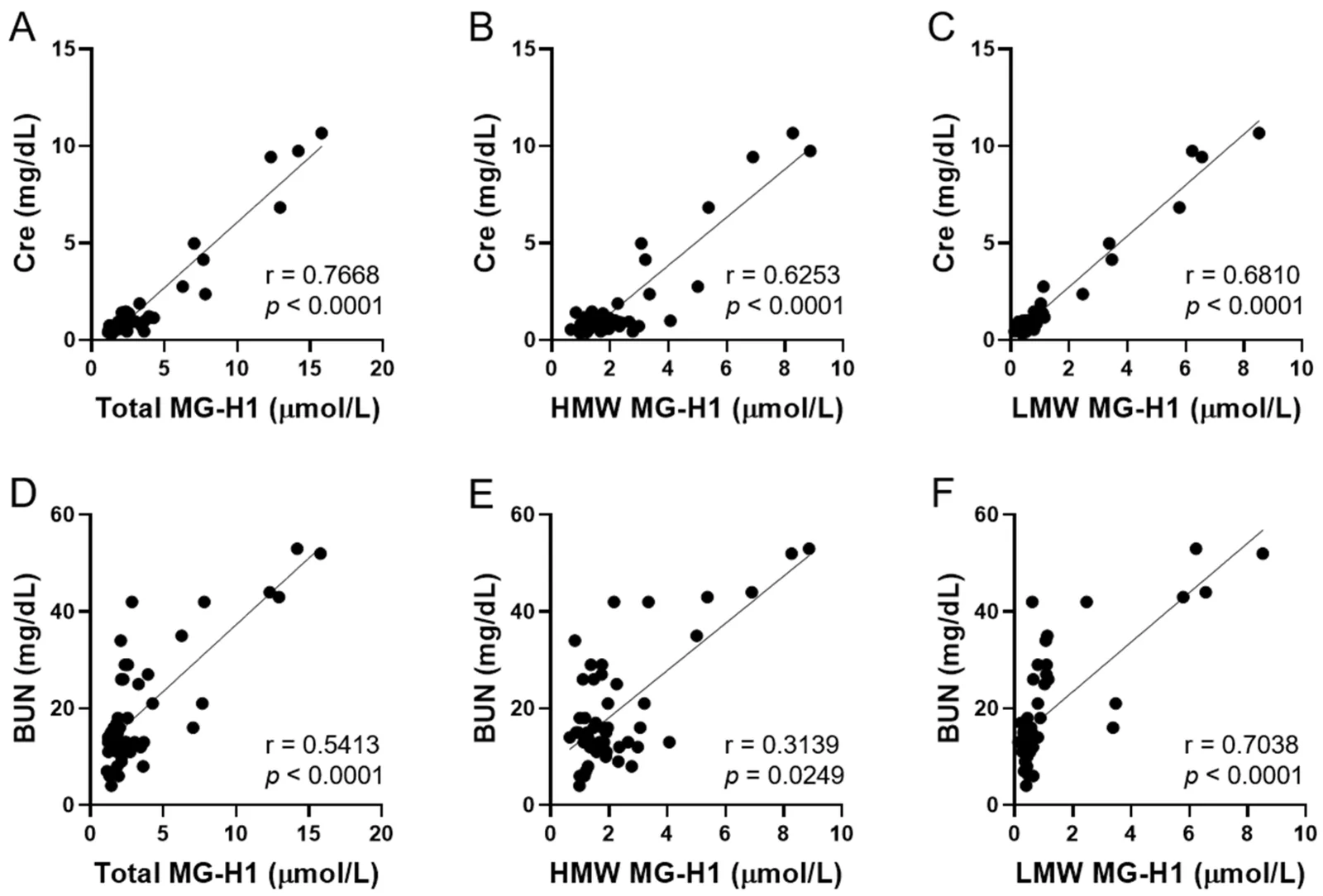
Correlation between MG-H1 levels and renal function markers. (**A**–**C**) Total MG-H1 (**A**), high-molecular-weight MG-H1 (HMW MG-H1, and (**B**)), low-molecular-weight MG-H1 (LMW MG-H1, (**C**)) are plotted against serum creatinine (Cre). (**D**–**F**) Total MG-H1 (**D**), HMW MG-H1 (**E**), and LMW MG-H1 (**F**) are plotted against blood urea nitrogen (BUN). Spearman’s correlation coefficients (r) and *p*-values are presented in each panel.

**Figure 4:**
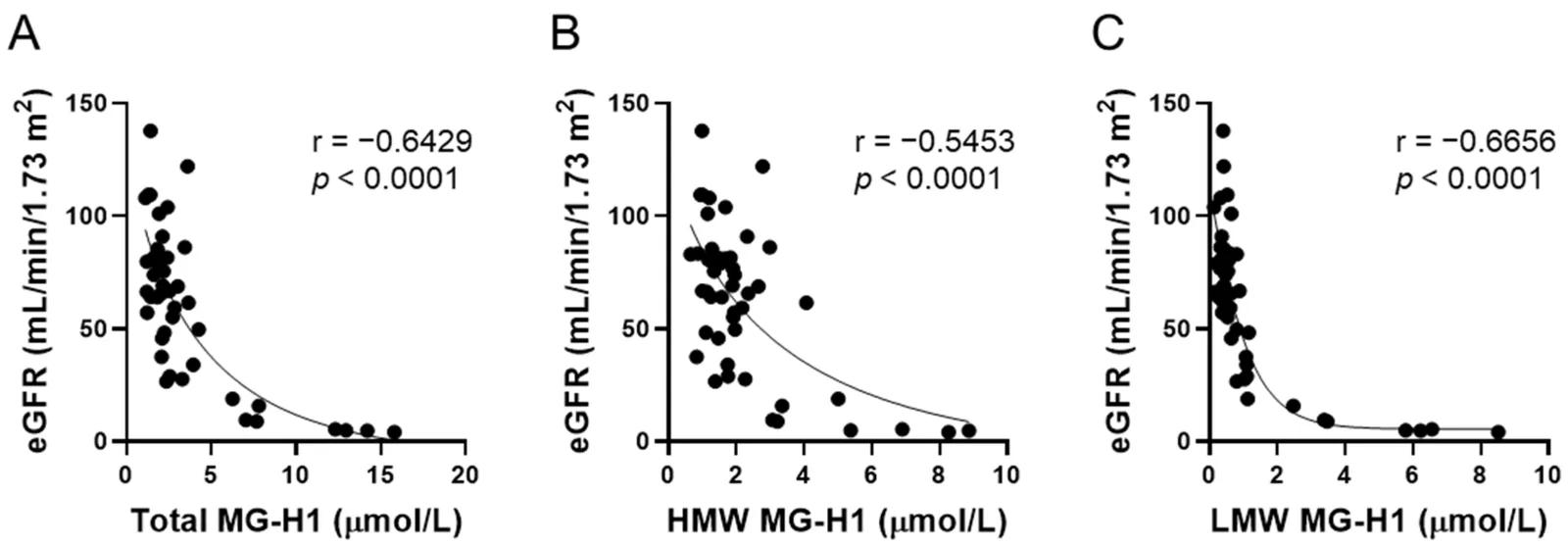
The correlation between MG-H1 levels and estimated glomerular filtration rate (eGFR). Total MG-H1 (**A**), high-molecular-weight MG-H1 (HMW MG-H1, (**B**)), and low-molecular-weight MG-H1 (LMW MG-H1, (**C**)) plotted against eGFR values to determine their associations. Spearman correlation coefficients (r) and *p*-values are presented in each panel.

To determine the relationship between MG-H1 levels and renal function, we determined the correlations between MG-H1 (total, HMW, and LMW) and renal biomarkers, including serum Cre and BUN. All MG-H1 forms exhibited a positive correlation with serum Cre levels (Figure 3A–C). The Spearman correlation coefficients were as follows: total MG-H1: r = 0.7668, *p* < 0.0001; HMW MG-H1: r = 0.6253, *p* < 0.0001; LMW MG-H1: r = 0.6810, *p* < 0.001. MG-H1 (total, HMW, and LMW) also showed a positive correlation with BUN levels (Figure 3D–F). The correlation coefficients were as follows: total MG-H1: r = 0.5413, *p* < 0.0001; HMW MG-H1: r = 0.3139, *p* = 0.0249; LMW MG-H1: r = 0.7038, *p* < 0.0001. Moreover, each MG-H1 form exhibited a significant correlation with eGFR and exhibited a negative association (Figure 4A–C). The correlation coefficients were as follows: total MG-H1: r = −0.6429, *p* < 0.0001; HMW MG-H1: r = −0.5453, *p* < 0.0001; LMW MG-H1: r = −0.6656, *p* < 0.0001. Although the correlation coefficients between each form of MG-H1 and renal function markers differed to some extent, the overall variation was minimal, indicating a consistent association across all MG-H1 forms.

**Figure 5:**
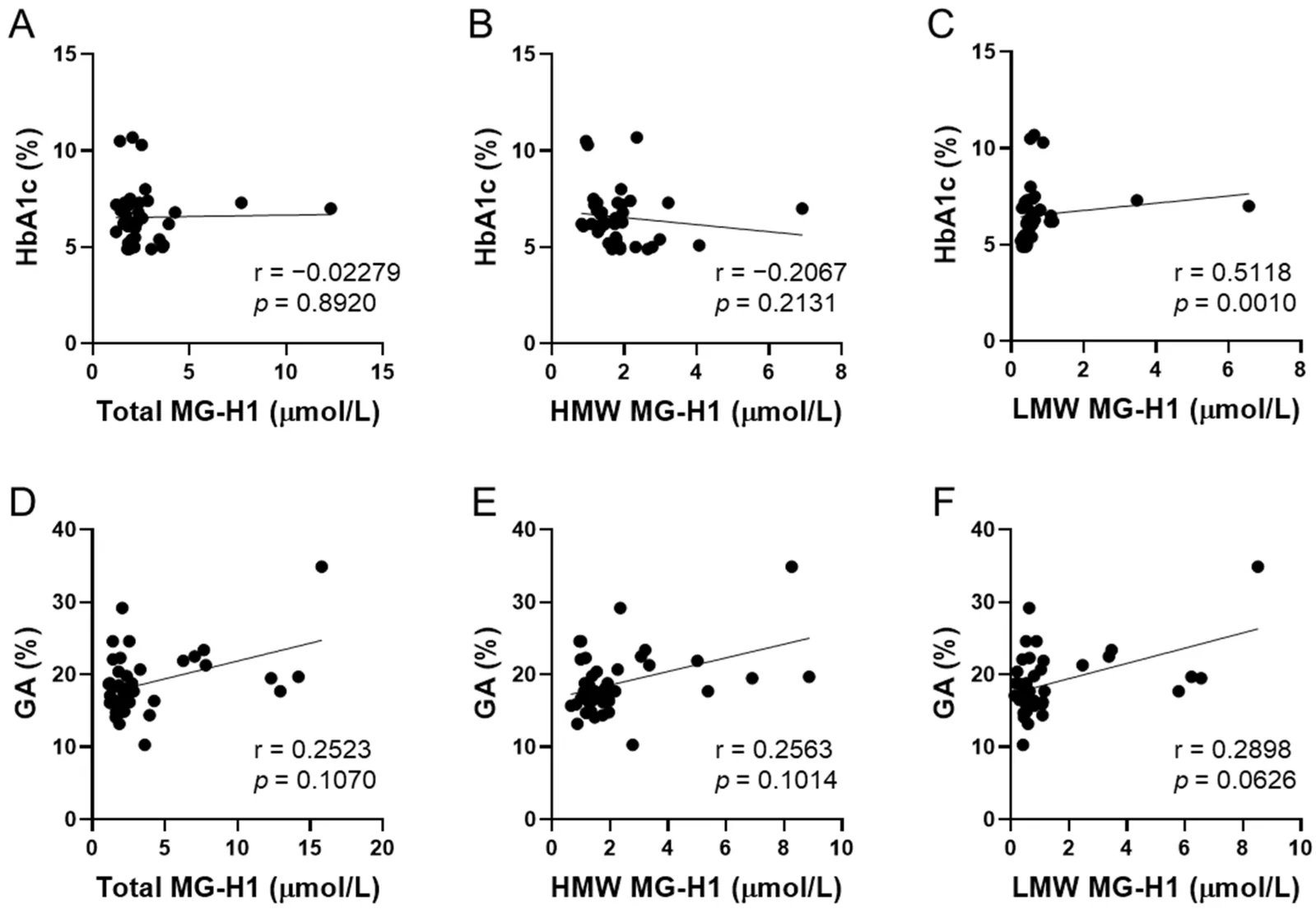
Correlation between MG-H1 levels and diabetes markers. (**A**–**C**) Total MG-H1 (**A**), high-molecular-weight MG-H1 (HMW MG-H1, (**B**)), and low-molecular-weight MG-H1 (LMW MG-H1, (**C**)) plotted against HbA1c levels. (**D**–**F**) Total MG-H1 (**D**), HMW MG-H1 (**E**), and LMW MG-H1 (**F**) plotted against glycoalbumin (GA) levels.

All MG-H1 forms exhibited no significant correlations with glycoalbumin (total MG-H1, r = 0.2523, *p* = 0.1070; HMW MG-H1, r = 0.2563, *p* = 0.1014; and LMW MG-H1, r = 0.2898, *p* = 0.0626; Figure 5). Although LMW MG-H1 showed a moderate positive correlation with HbA1c (r = 0.5118, *p* = 0.0010), no significant correlations were observed for total or HMW MG-H1. The lack of association with glycoalbumin suggests that serum albumin may not be a major target of MG-H1 modification in circulation. Furthermore, to assess the potential association between MG-H1 and systemic inflammation, we examined the correlations between C-reactive protein (CRP) levels and MG-H1s. As presented in Supplementary Figure S3, no significant correlations were observed between any MG-H1 form and CRP, one of the inflammatory markers evaluated in this study.

### 3.5. ROC Curve Analysis of MG-H1 and Conventional Renal Function Markers for DKD Diagnosis

**Figure 6:**
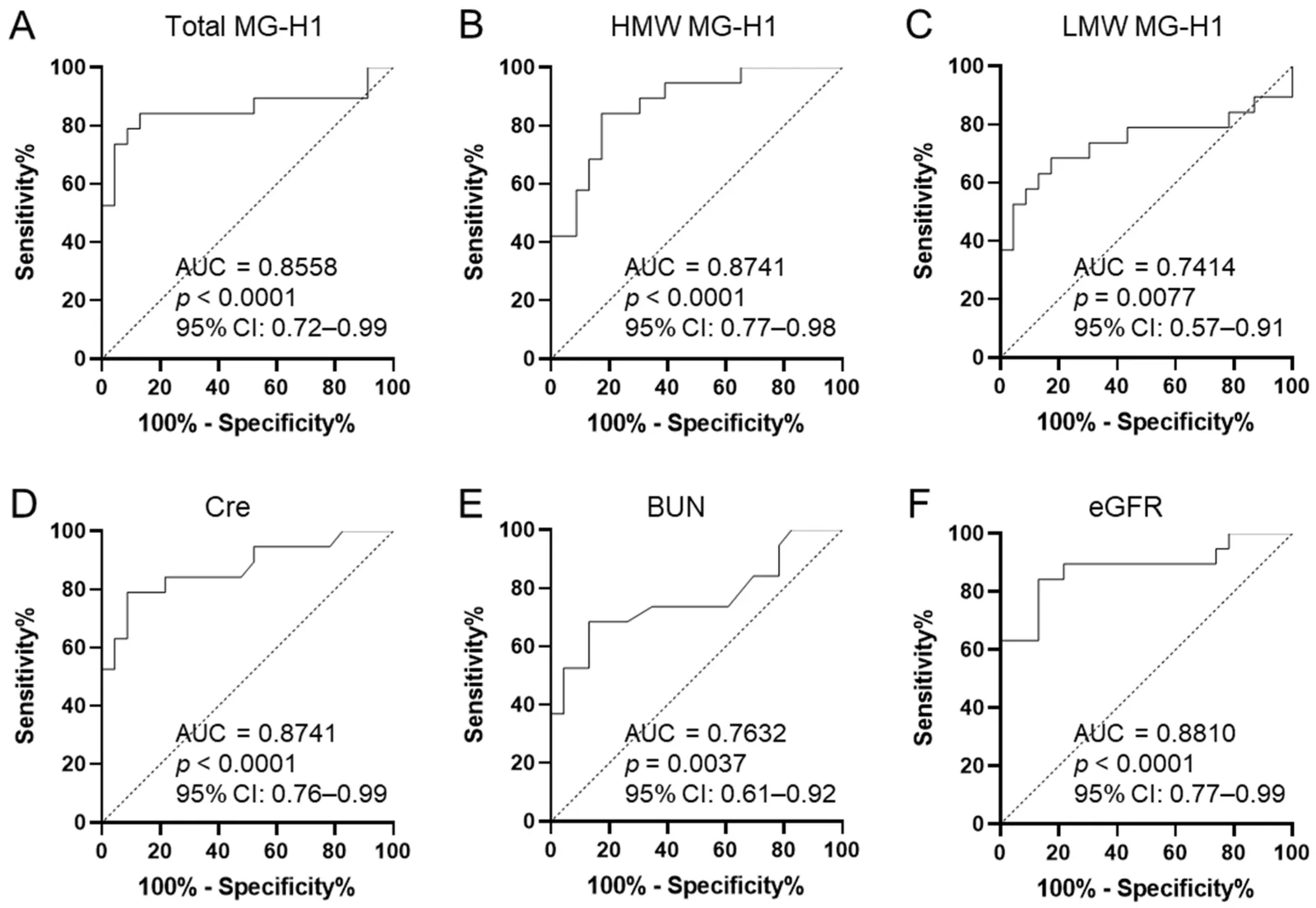
Receiver operating characteristic (ROC) curve analysis for the diagnostic performance of MG-H1 and conventional renal function markers to distinguish diabetic kidney disease (DKD) from patients with diabetes without kidney disease (DM). (**A**–**C**) ROC curves for total MG-H1 (**A**), high-molecular-weight MG-H1 (HMW MG-H1, (**B**)), and low-molecular-weight MG-H1 (LMW MG-H1, (**C**)). (**D**–**F**) ROC curves for serum creatinine (Cre, (**D**)), blood urea nitrogen (BUN, (**E**)), and estimated glomerular filtration rate (eGFR, (**F**)). The area under the curve (AUC) values, *p*-values, and 95% confidence intervals (95% CIs) are shown in each panel.

To evaluate the potential diagnostic performance of each form of MG-H1 for distinguishing DKD from DM, we conducted ROC curve analyses using data from patients with DM and DKD (Figure 6A–C). The AUCs (95% CIs) for total, HMW, and LMW MG-H1 were 0.8558 (0.72–0.99, *p* < 0.0001), 0.8741 (0.77–0.98, *p* < 0.0001), and 0.7414 (0.57–0.91, *p* = 0.0077), respectively. Among the three MG-H1 forms, HMW MG-H1 exhibited the highest diagnostic performance for discriminating DKD from DM, followed closely by total MG-H1. Although LMW MG-H1 also showed significant discriminative ability, its diagnostic performance was lower than that of total and HMW MG-H1. To compare the diagnostic performance of MG-H1 with conventional renal function markers, ROC curve analyses were also performed using serum Cre, BUN, and eGFR (Figure 6D–F). The AUCs (95% CIs) for conventional renal function markers were 0.8741 (0.76–0.99, *p* < 0.0001) for Cre, 0.7632 (0.61–0.92, *p* = 0.0037) for BUN, and 0.8810 (0.77–0.99, *p* < 0.0001) for eGFR. DeLong test was used to compare the AUCs of the ROC curves. Significant differences were identified between LMW MG-H1 and Cre (*p* = 0.0055), LMW MG-H1 and eGFR (*p* = 0.0153), BUN and Cre (*p* = 0.0412), and BUN and eGFR (*p* = 0.0350). None of the other pairwise comparisons reached statistical significance (between total MG-H1 and Cre [*p* = 0.782], between total MG-H1 and BUN [*p* = 0.168], between total MG-H1 and eGFR [*p* = 0.74], between HMW MG-H1 and Cre [*p* = 1.000], between HMW MG-H1 and BUN [*p* = 0.173], between HMW MG-H1 and eGFR [*p* = 0.919], or between LMW MG-H1 and BUN [*p* = 0.667]). Although some differences were observed among the MG-H1 forms, total and HMW MG-H1 demonstrated diagnostic performance comparable to that of conventional renal function markers. These findings suggest that MG-H1, particularly its total and HMW forms, may be useful for identifying DKD in patients with diabetes and may provide information complementary to established renal function markers. However, given the limited sample size and pilot nature of this study, these ROC analyses were conducted as exploratory assessments of discriminatory performance. Accordingly, clinically applicable diagnostic thresholds and sensitivity, specificity, and validation metrics were not established and require evaluation in larger independent cohorts.

## 4. Discussion

This study developed a monoclonal antibody-based ELISA for MG-H1 and applied it to a pilot cohort to explore clinical associations. The antibody showed high specificity for MG-H1 with minimal cross-reactivity to structurally related AGEs and precursors, supporting its utility in quantifying MG-H1 in human serum. Another important feature of this ELISA system is its ability to independently quantify LMW, HMW, and total MG-H1. This distinction may be clinically relevant because each form exhibits unique diagnostic performance for DKD. To the best of our knowledge, no commercially available ELISA kits currently offer this capability, which emphasizes the novelty and additional diagnostic utility of our assay.

MG-H1 predominantly exists in serum in a protein-bound form. It is accompanied by low amounts of LMW peptide-bound MG-H1 in various configurations [11]. For LC–MS/MS analysis, serum MG-H1 is fractionated by molecular weight cut-off filtration into HMW and LMW forms. The latter is further processed to hydrolyze the peptide-bound form into free MG-H1 [14,27]. The HMW fraction reflects long-term glycation of structural and circulating proteins, whereas the LMW fraction, released during proteolytic degradation of glycated proteins, is primarily excreted through the kidneys [14,28]. Measurement of these MG-H1 forms was shown to be applicable not only for assessing renal function but also for evaluating the risk of cardiovascular and neurodegenerative diseases [16,28].

Our results demonstrate that MG-H1 levels are increased in patients with DKD and are closely associated with renal dysfunction. Although the magnitude of the changes varied among the different MG-H1 forms, the DKD group consistently exhibited the highest levels of total, HMW, and LMW MG-H1 among the study groups. These findings are consistent with previous studies showing that MG-H1 accumulation reflects renal metabolic stress and impaired clearance mechanisms and further support the association between circulating MG-H1 levels and renal dysfunction [14,28].

MG-H1 levels were strongly correlated with conventional renal function parameters. Total, HMW, and LMW MG-H1 levels were positively correlated with serum Cre and BUN, and negatively correlated with eGFR (Figure 3 and Figure 4). These results are consistent with previous studies indicating that AGEs, including MG-H1, may accumulate in renal tissues and contribute to nephropathy through oxidative stress, inflammation, and fibrosis [28]. In contrast, none of the MG-H1 forms showed a significant correlation with glycoalbumin, while only LMW MG-H1 exhibited a significant positive correlation with HbA1c (Figure 5). Although this result was unexpected, it suggests that serum albumin, despite being an abundant protein, is not the primary target of MG-H1 modification. Instead, other proteins that are more susceptible to reacting with MGO are likely involved. This observation is consistent with a proteome-wide analysis using a chemical probe reported by Sibbersen et al. [29].

Our ROC analysis showed that all three MG-H1 forms significantly discriminated patients with DKD from diabetic patients without kidney disease. Among these forms, HMW MG-H1 showed the highest diagnostic performance, closely followed by total MG-H1, whereas LMW MG-H1 had lower but still significant discriminatory ability. These results suggest that circulating MG-H1, particularly its HMW form, may be useful for identifying renal involvement in patients with diabetes. Consistent with previous LC–MS/MS-based studies, MG-H1 levels were found to be closely associated with renal dysfunction, further supporting the clinical relevance of MG-H1 as a marker of altered glycation and impaired renal clearance [14]. Relative to conventional renal function markers, MG-H1 generally showed comparable diagnostic performance, although differences were observed among the individual MG-H1 forms. Collectively, these findings support the potential utility of MG-H1 as a DKD biomarker and suggest that its measurement may provide information complementary to established renal biomarkers. However, given the relatively small pilot cohort in the present study, the reported ROC analyses and estimates of diagnostic performance should be considered preliminary. Larger, independent cohorts will be required to validate these findings, assess their robustness, and establish the clinical utility of MG-H1.

MG-H1 is implicated in DKD pathogenesis through the activation of RAGE, which promotes inflammatory and fibrotic pathways in renal tissues [30]. MG-H1 exhibits higher binding affinity for RAGE than for other AGEs, e.g., CML and CEL [31]. Moreover, MG-H1-modified albumin, which mimics the HMW form, can bind to RAGE and activate downstream signaling, suggesting that MG-H1 is a primary and early mediator in AGE–RAGE-related pathophysiology [31]. Because of the involvement of AGEs in various chronic diseases, including cardiovascular disease, neurodegenerative disorders, and diabetic complications, monitoring circulating MG-H1 levels might be useful for predictive and preventive medicine. However, the predictive and prognostic value of MG-H1 was not assessed in this study and requires investigation in prospective longitudinal studies.

In addition to renal dysfunction, oxidative stress may contribute to MG-H1 accumulation in diabetes. Increased oxidative and dicarbonyl stress can promote the formation of reactive carbonyl compounds such as MGO, thereby accelerating MG-H1 generation and the formation of other AGEs. Previous experimental studies have shown that modulation of oxidative stress can improve glycemic status and attenuate oxidative imbalance in diabetic models, further supporting the close relationship between hyperglycemia, oxidative stress, and AGE formation [32]. Thus, circulating MG-H1 levels may reflect not only impaired renal clearance but also broader metabolic processes related to oxidative stress.

Age is an important consideration when interpreting circulating AGE levels because AGE accumulation generally increases with aging. In this study, the Ctrl group was significantly younger than the DM and DKD groups. However, the DM and DKD groups did not differ significantly in age (Table 1), suggesting that age alone is unlikely to explain the higher MG-H1 levels in patients with DKD. Nevertheless, a potential effect of age cannot be completely excluded, and larger studies using age-matched cohorts and multivariable analyses are required to clarify the independent contribution of renal dysfunction to circulating MG-H1 levels. In addition, multivariate regression models adjusted for potential confounding factors, such as age, sex, glycemic control, and renal function, should be used to evaluate the independent diagnostic value of MG-H1.

MG-H1 should also be considered in the broader context of AGE biomarkers. Several AGEs, including Nε-carboxymethyllysine (CML), Nε-carboxyethyllysine (CEL), pentosidine, and MG-H1, have been investigated as markers of glycation-related tissue damage and disease progression [5]. Among these AGEs, MG-H1 is one of the most abundant MGO-derived adducts [11] and has been implicated in the pathogenesis of diabetic complications through RAGE-mediated signaling pathways [31]. Thus, MG-H1 may complement established AGE biomarkers by providing additional information on dicarbonyl stress and AGE accumulation associated with renal dysfunction.

This study has several limitations that constrain the generalizability of the findings including a small sample size, single-center and Japan-centric recruitment, a lack of external validation, and incomplete analytical validation. Although intra-assay precision was evaluated, inter-assay precision was not assessed. While the mass balance between total MG-H1 and the sum of HMW and LMW MG-H1 indicated no apparent systematic loss during fractionation, variability among samples was observed, and filter adsorption and cross-contamination between fractions were not formally assessed. In addition, the commutability of the free MG-H1 calibrator with endogenous MG-H1 species was not assessed, and differences in epitope accessibility among the MG-H1 molecular forms may affect absolute quantification. Therefore, further analytical validation of the assay and filtration procedure is warranted.

Clinical group classification was based on diagnoses recorded in the biobank rather than an independently adjudicated reference standard, and conventional renal biomarkers may have contributed to the diagnosis of DKD. Furthermore, the use of anonymized residual serum samples limited access to detailed clinical information, including CKD stage, albuminuria status, diabetes duration, and the exclusion of non-diabetic kidney disease, thereby precluding stage-specific and etiology-based analyses. Correlation analyses were not adjusted for potential confounders (e.g., age, sex, and diabetes duration), and multiple comparisons were performed without formal correction, increasing the risk of type I error. Consequently, the observed associations and ROC-based estimates of diagnostic performance should be considered exploratory and require validation in larger, independent, and more diverse cohorts. Future studies should incorporate multivariable and stage-specific analyses, evaluate assay reproducibility across sites, and establish clinical decision thresholds and reference intervals to support clinical implementation.

## 5. Conclusions

We developed a novel ELISA system capable of quantifying MG-H1 in its total, HMW, and LMW forms. In this pilot study, all MG-H1 forms were associated with renal dysfunction, and exploratory ROC analyses suggested that total and HMW MG-H1 may have the potential to discriminate patients with DKD from diabetic patients without kidney disease. These findings support the potential utility of MG-H1 as a biomarker of DKD; however, larger studies are required before its diagnostic utility can be established.

## Figures and Tables

**Table 1:** Demographic and clinical characteristics of the study participants.

Variable	Unit	*n* (Ctrl/DM/DKD)	Ctrl	DM	DKD	*p* (Overall)	*p* (Ctrl vs. DM)	*p* (Ctrl vs. DKD)	*p* (DM vs. DKD)
Age	years	10/23/19	53.8 ± 5.14	70.43 ± 11.47	72.11 ± 11.82	<0.0001	<0.0001	<0.0001	0.9544
Sex, male (%)	-	10/23/19	10 (100%)	10 (43.48%)	9 (47.37%)	0.0071	0.0022	0.005	>0.9999
HbA1c	%	10/18/10	5.13 ± 0.23	7.14 ± 1.67	6.93 ± 0.58	<0.0001	0.0003	<0.0001	0.9484
Glucose	mg/dL	10/19/14	95.6 ± 4.35	132.8 ± 41.0	142.7 ± 31.3	0.001	0.0028	0.0002	0.8156
Cre	mg/dL	9/23/19	0.85 ± 0.11	0.68 ± 0.25	3.29 ± 3.40	0.0008	0.0341	0.0173	0.0108
BUN	mg/dL	9/23/19	12.44 ± 2.24	14.78 ± 6.91	28.16 ± 14.53	<0.0001	0.4	0.0005	0.0033
eGFR	mL/min/1.73 m^2^	9/23/19	75.43 ± 10.13	82.56 ± 23.96	35.10 ± 29.58	<0.0001	0.5638	<0.0001	<0.0001

Continuous variables are presented as mean ± standard deviation (SDs), whereas categorical variables are presented as *n* (%). Differences in continuous variables among groups were assessed using Welch’s ANOVA with the Brown–Forsythe correction for unequal variances and sample sizes. Pairwise group comparisons were conducted using the Games–Howell method with Holm adjustment for multiple testing. Sex distributions were assessed using the chi-square test; pairwise group comparisons were conducted using Fisher’s exact test with Holm correction. Ctrl, healthy controls; DM, diabetes without kidney disease; DKD, diabetic kidney disease; Cre, creatinine; BUN, blood urea nitrogen; eGFR, estimated glomerular filtration rate.

**Table 2:** Recovery rates of MG-H1 in human serum samples (*n* = 3).

Spiked Conc. (μmol/L)	MG-H1 (μmol/L) ^1^	CV% ^2^	Recovery (%) ^1^
0	2.24 ± 0.08	3.74	-
2.5	5.15 ± 0.18	3.40	108.85 ± 5.23
5.0	8.04 ± 0.73	9.10	111.11 ± 11.19
10.0	14.52 ± 0.22	1.52	118.64 ± 2.23

^1^ Values are expressed as mean ± standard deviation (SD). ^2^ CV: coefficient of variation.

## Data Availability

The raw data supporting the conclusions of this article will be made available by the authors on request.

## Supplementary Materials

The following supporting information can be downloaded at: https://www.mdpi.com/article/10.3390/diagnostics16162593/s1, Figure S1: Calibration curve for MG-H1 generated from ELISA; Figure S2: Interference testing for ELISA; Figure S3: The correlation between MG-H1 levels and C-reactive protein (CRP).

## Abbreviations

The following abbreviations are used in this manuscript:

AGE	Advanced glycation end product
AUC	Area under the ROC curve
BUN	Blood urea nitrogen
CKD	Chronic kidney disease
Cre	Creatinine
DKD	Diabetic kidney disease
eGFR	Estimated glomerular filtration rate
ELISA	Enzyme-linked immunosorbent assay
HMW	High-molecular-weight
LMW	Low-molecular-weight
MG-H1	Methylglyoxal-derived hydroimidazolone-1
MGO	Methylglyoxal
RAGE	Receptor for advanced glycation endproduct
RECHS	Resource Center for Health Science
ROC	Receiver operating characteristic
SD	Standard deviation

## References

[1] Rabbani N., Thornalley P.J. (2018). Advanced glycation end products in the pathogenesis of chronic kidney disease. Kidney Int..

[2] Adeshara K., Gordin D., Antikainen A.A., Harjutsalo V., Sandholm N., Lehto M.J., Groop P.H. (2024). Protein glycation products associate with progression of kidney disease and incident cardiovascular events in individuals with type 1 diabetes. Cardiovasc. Diabetol..

[3] Schwedler S.B., Metzger T., Schinzel R., Wanner C. (2002). Advanced glycation end products and mortality in hemodialysis patients. Kidney Int..

[4] Goh S.Y., Cooper M.E. (2008). Clinical review: The role of advanced glycation end products in progression and complications of diabetes. J. Clin. Endocrinol. Metab..

[5] Schalkwijk C.G., Stehouwer C.D.A. (2020). Methylglyoxal, a Highly Reactive Dicarbonyl Compound, in Diabetes, Its Vascular Complications, and Other Age-Related Diseases. Physiol. Rev..

[6] Li J., Liu D., Sun L., Lu Y., Zhang Z. (2012). Advanced glycation end products and neurodegenerative diseases: Mechanisms and perspective. J. Neurol. Sci..

[7] Raghavan C.T. (2024). Advanced Glycation End Products in Neurodegenerative Diseases. J. Mol. Neurosci..

[8] Rabbani N., Thornalley P.J. (2014). The critical role of methylglyoxal and glyoxalase 1 in diabetic nephropathy. Diabetes.

[9] Ishibashi Y., Matsui T., Nakamura N., Sotokawauchi A., Higashimoto Y., Yamagishi S.I. (2017). Methylglyoxal-derived hydroimidazolone-1 evokes inflammatory reactions in endothelial cells via an interaction with receptor for advanced glycation end products. Diabetes Vasc. Dis. Res..

[10] Huang S.F., Chang Y.T., Hsia S.M., Liao K.W., Tsai C.Y., Huang S.Y., Ho C.T., Hung W.L. (2025). Distinct effects of methylglyoxal-derived hydroimidazolone 1, N(ε)-carboxyethyllysine, and an advanced glycation end product-rich diet on lipid metabolism, gut microbiota, and secondary bile acids in high-fat diet-induced obese mice. Food Chem..

[11] Rabbani N., Thornalley P.J. (2014). Dicarbonyl proteome and genome damage in metabolic and vascular disease. Biochem. Soc. Trans..

[12] Oliveira A.L., de Oliveira M.G., Mónica F.Z., Antunes E. (2024). Methylglyoxal and Advanced Glycation End Products (AGEs): Targets for the Prevention and Treatment of Diabetes-Associated Bladder Dysfunction?. Biomedicines.

[13] Kim G., Yoo H.J., Yoo M.K., Choi J.H., Lee K.W. (2024). Methylglyoxal-derived hydroimidazolone-1/RAGE axis induces renal oxidative stress and renal fibrosis In Vitro and In Vivo. Toxicology.

[14] Rabbani N., Adaikalakoteswari A., Larkin J.R., Panagiotopoulos S., MacIsaac R.J., Yue D.K., Fulcher G.R., Roberts M.A., Thomas M., Ekinci E. (2022). Analysis of Serum Advanced Glycation Endproducts Reveals Methylglyoxal-Derived Advanced Glycation MG-H1 Free Adduct Is a Risk Marker in Non-Diabetic and Diabetic Chronic Kidney Disease. Int. J. Mol. Sci..

[15] Martens R.J.H., Broers N.J.H., Canaud B., Christiaans M.H.L., Cornelis T., Gauly A., Hermans M.M.H., Konings C., van der Sande F.M., Scheijen J. (2019). Relations of advanced glycation endproducts and dicarbonyls with endothelial dysfunction and low-grade inflammation in individuals with end-stage renal disease in the transition to renal replacement therapy: A cross-sectional observational study. PLoS ONE.

[16] Ito K., Sakata N., Nagai R., Shirakawa J.I., Watanabe M., Mimata A., Abe Y., Yasuno T., Sasatomi Y., Miyake K. (2017). High serum level of methylglyoxal-derived AGE, Nδ-(5-hydro-5-methyl-4-imidazolone-2-yl)-ornithine, independently relates to renal dysfunction. Clin. Exp. Nephrol..

[17] Oshima M., Shimizu M., Yamanouchi M., Toyama T., Hara A., Furuichi K., Wada T. (2021). Trajectories of kidney function in diabetes: A clinicopathological update. Nat. Rev. Nephrol..

[18] Sanajou D., Ghorbani Haghjo A., Argani H., Aslani S. (2018). AGE-RAGE axis blockade in diabetic nephropathy: Current status and future directions. Eur. J. Pharmacol..

[19] Nakamura T., Tsujimoto T., Yasuda K., Kajio H., Ueki K. (2025). Consistently low serum levels of MG-H1 is associated with a lower risk of diabetic kidney disease. J. Clin. Endocrinol. Metab..

[20] Bora S., Adole P.S., Motupalli N., Pandit V.R., Vinod K.V. (2020). Association between carbonyl stress markers and the risk of acute coronary syndrome in patients with type 2 diabetes mellitus-A pilot study. Diabetes Metab. Syndr. Clin. Res. Rev..

[21] Šebeková K., Kovalčíková A.G., Čagalová A., Tichá Ľ., Podracká Ľ. (2025). Association of plasma levels of protein-bound advanced glycation end-products and their soluble receptors with bone mineral status in young girls with the restrictive type of anorexia nervosa. Arch. Osteoporos..

[22] Morishita T., Takemura M., Hayashi M., Saito K., Yamamoto Y., Tsurumi H., Matsunami H. (2024). Cohort profile: Rationale and design of the Resource Center for Health Science (RECHS) project—A study of health hazards and medical cost burden among the Japanese population. BMJ Open.

[23] Yamaguchi H., Nagai M., Sugawa H., Yasuda H., Nagai R. (2021). Development of a conventional immunochemical detection system for determination of N(delta)-(5-hydro-5-methyl-4-imidazolone-2-yl)-ornithine in methylglyoxal-modified proteins. Glycoconj. J..

[24] Wang T., Streeter M.D., Spiegel D.A. (2015). Generation and characterization of antibodies against arginine-derived advanced glycation endproducts. Bioorg. Med. Chem. Lett..

[25] Welch N.G., Scoble J.A., Muir B.W., Pigram P.J. (2017). Orientation and characterization of immobilized antibodies for improved immunoassays (Review). Biointerphases.

[26] Klopfer A., Spanneberg R., Glomb M.A. (2011). Formation of arginine modifications in a model system of Nalpha-tert-butoxycarbonyl (Boc)-arginine with methylglyoxal. J. Agric. Food Chem..

[27] Wang W., Zhu Y., Sang S. (2025). Optimization for Simultaneous Determination of a Panel of Advanced Glycation End Products as Biomarkers for Metabolic Diseases. J. Agric. Food Chem..

[28] Vangrieken P., Scheijen J., Schiffers P.M.H., van de Waarenburg M.P.H., Foulquier S., Schalkwijk C.C.G. (2025). Modelling the effects of elevated methylglyoxal levels on vascular and metabolic complications. Sci. Rep..

[29] Sibbersen C., Schou Oxvig A.M., Bisgaard Olesen S., Nielsen C.B., Galligan J.J., Jørgensen K.A., Palmfeldt J., Johannsen M. (2018). Profiling of Methylglyoxal Blood Metabolism and Advanced Glycation End-Product Proteome Using a Chemical Probe. ACS Chem. Biol..

[30] Taguchi K., Fukami K. (2023). RAGE signaling regulates the progression of diabetic complications. Front. Pharmacol..

[31] Xue J., Ray R., Singer D., Böhme D., Burz D.S., Rai V., Hoffmann R., Shekhtman A. (2014). The receptor for advanced glycation end products (RAGE) specifically recognizes methylglyoxal-derived AGEs. Biochemistry.

[32] Teofilović B., Tomas A., Martić N., Stilinović N., Čapo I., Grujić-Letić N., Gligorić E., Rašković A. (2025). Therapeutic potential of basil (*Ocimum basilicum* L.) aqueous extract: Impact on glycemia and oxidative stress in normoglycemic and diabetic rats. Pharmazie.

