# Supplementary material for: Monoclonal Antibody-Based ELISA Quantification of Serum Methylglyoxal-Derived Hydroimidazolone-1
Source: Diagnostics (Basel). 2026 Aug 16;16(16):2593. doi: 10.3390/diagnostics16162593 (PMC13512253; doi:10.3390/diagnostics16162593)
Supplement: Supplementary file 1 [file diagnostics-16-02593-s001.zip › diagnostics-4489457-supplementary.pdf]

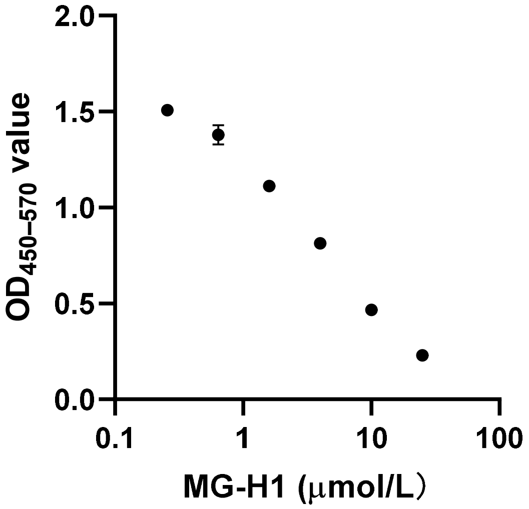

Supplementary Figure S1. Calibration curve for MG-H1 generated from ELISA. The curve demonstrates linearity across the concentration range of 0.26–25.0 μmol/L, with a coefficient of determination (R^2) of 0.99. Data represent the mean ± standard deviation (SD) of three independent measurements.

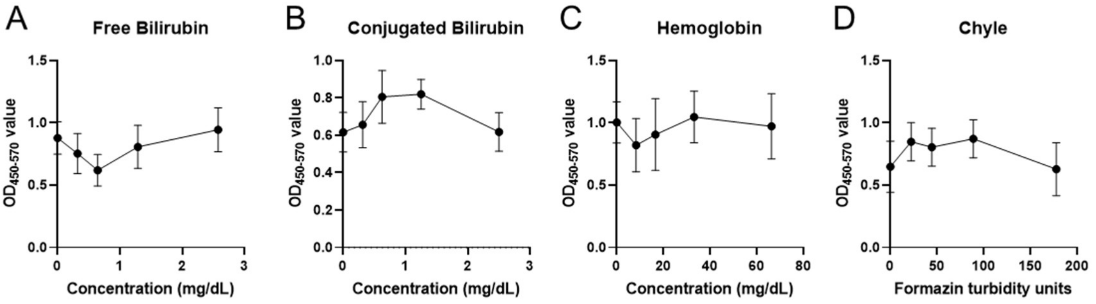

Supplementary Figure S2. Interference testing for ELISA. Serum samples were spiked with potential endogenous substances. Free bilirubin was tested at 0, 0.32, 0.64, 1.29, and 2.58 mg/dL (A); conjugated bilirubin at 0, 0.31, 0.63, 1.25, and 2.50 mg/dL (B); hemoglobin at 0, 8.28, 16.56, 33.13, and 66.25 mg/dL (C); and chyle at 0, 22.19, 44.38, 88.75, and 177.50 formazin turbidity units (D). OD_{450} values were measured, and data are presented as mean ± SD of six replicate samples.

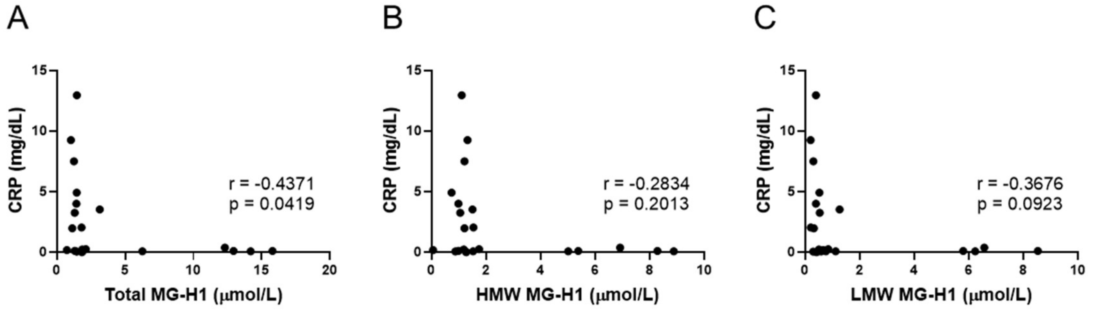

Supplementary Figure S3. The correlation between MG-H1 levels and C-reactive protein (CRP). Total MG-H1 (A), high-molecular-weight MG-H1 (HMW MG-H1, B), and low-molecular-weight MG-H1 (LMW MG-H1, C) were plotted against CRP values to determine their associations. Spearman's correlation coefficients (r) and p-values are shown in each panel.
